# Deep Mutational Scanning Reveals the Active-Site Sequence Requirements for the Colistin Antibiotic Resistance Enzyme MCR-1

**DOI:** 10.1128/mBio.02776-21

**Published:** 2021-11-16

**Authors:** Zhizeng Sun, Timothy Palzkill

**Affiliations:** a Department of Pharmacology and Chemical Biology, Baylor College of Medicine, Houston, Texas, USA; McMaster University

**Keywords:** antibiotic resistance, colistin resistance, enzyme kinetics, enzyme purification, mutagenesis, polymyxins

## Abstract

Colistin (polymyxin E) and polymyxin B have been used as last-resort agents for treating infections caused by multidrug-resistant Gram-negative bacteria. However, their efficacy has been challenged by the emergence of the mobile colistin resistance gene *mcr-1*, which encodes a transmembrane phosphoethanolamine (PEA) transferase enzyme, MCR-1. The enzyme catalyzes the transfer of the cationic PEA moiety of phosphatidylethanolamine (PE) to lipid A, thereby neutralizing the negative charge of lipid A and blocking the binding of positively charged polymyxins. This study aims to facilitate understanding of the mechanism of the MCR-1 enzyme by investigating its active-site sequence requirements. For this purpose, 23 active-site residues of MCR-1 protein were randomized by constructing single-codon randomization libraries. The libraries were individually selected for supporting Escherichia coli cell growth in the presence of colistin or polymyxin B. Deep sequencing of the polymyxin-resistant clones revealed that wild-type residues predominates at 17 active-site residue positions, indicating these residues play critical roles in MCR-1 function. These residues include Zn^2+^-chelating residues as well as residues that may form a hydrogen bond network with the PEA moiety or make hydrophobic interactions with the acyl chains of PE. Any mutations at these residues significantly decrease polymyxin resistance levels and the PEA transferase activity of the MCR-1 enzyme. Therefore, deep sequencing of the randomization libraries of MCR-1 enzyme identifies active-site residues that are essential for its polymyxin resistance function. Thus, these residues may be utilized as targets to develop inhibitors to circumvent MCR-1-mediated polymyxin resistance.

## INTRODUCTION

**FIG 1:**
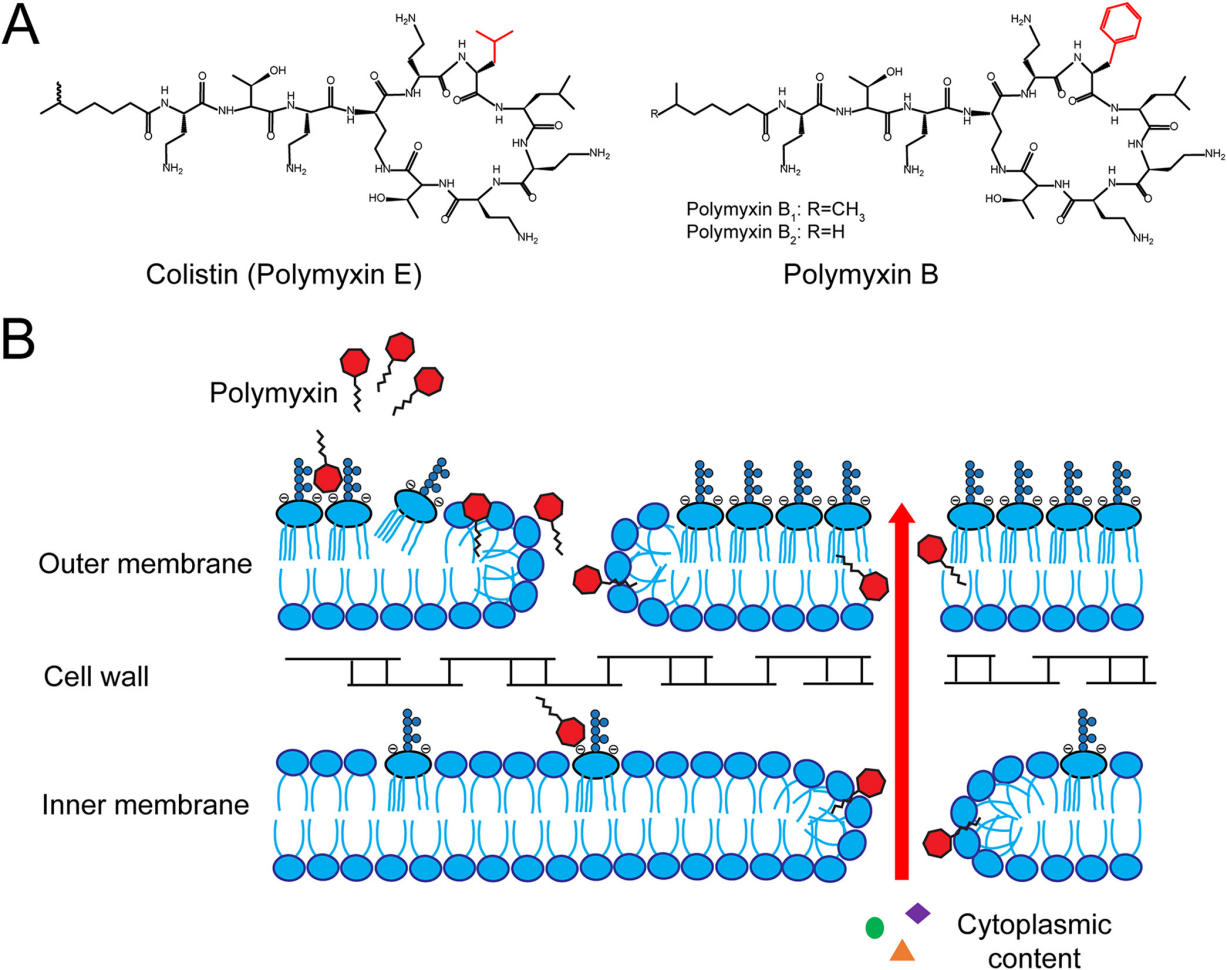
Chemical structures and mechanism of action of polymyxins. (A) Chemical structures of colistin (polymyxin E) and polymyxin B. The amino acid residue that is different between colistin and polymyxin B is labeled red. (B) Diagram depicting bactericidal action of polymyxin by targeting lipopolysaccharide on outer membrane and inner membrane Gram-negative bacteria.

Polymyxins are a group of cationic antimicrobial peptides produced in nature by the Gram-positive bacterium Paenibacillus polymyxa (1). They are lipopeptides consisting of a cyclic heptapeptide ring with three positively charged amine groups, a tail tripeptide moiety with two positively charged amine groups, and a hydrophobic acyl chain tail (Fig. 1) (2). Polymyxins are bactericidal against Gram-negative bacteria, and their initial binding target is the lipopolysaccharide (LPS) component of the outer membrane of Gram-negative bacteria. They bind to lipid A of the LPS through electrostatic interactions between their positively charged amine groups and the negatively charged phosphate groups of lipid A. This interaction results in insertion of the hydrophobic fatty acyl chain and hydrophobic domain of the cyclic heptapeptide ring of polymyxins into the outer membrane and formation of pore-like aggregates on the outer membrane, which leads to permeabilization of the outer membrane. Subsequently, the polymyxin molecule inserts into and disrupts the physical integrity of the phospholipid bilayer of the inner membrane. This causes lysis of the inner membrane, leakage of intracellular contents, and cell death (2). In addition, recent studies suggest that colistin damages the inner membrane by acting on LPS at the inner membrane before the LPS is transported to the outer membrane (3). Besides permeabilizing the inner and outer membranes, polymyxins have also been shown to display a secondary mode of action by inhibiting the vital respiratory enzyme NADH-quinone oxidoreductase in the inner membrane of Gram-negative bacteria (4).

Polymyxins include a group of 5 chemically different compounds (polymyxins A to E). Polymyxin B and polymyxin E (colistin), which differ by only one amino acid in the cyclic heptapeptide ring (d-phenylalanine in polymyxin B versus d-leucine in colistin), are the only two polymyxins used clinically (Fig. 1) (5). Colistin and polymyxin B were originally introduced for clinical use in the 1950s for the treatment of infections caused by Gram-negative organisms. However, they were largely replaced by other antibiotics by the mid-1970s because of their high rate of nephrotoxicity and neurotoxicity. By the mid-1990s, colistin and polymyxin B were reintroduced into clinical practice due to the emergence of multidrug-resistant Gram-negative bacteria, which are resistant to nearly all other antibiotics (6). More recently, colistin and polymyxin B were used as last-line antibiotics in treating infections caused by multidrug-resistant Gram-negative bacteria, such as Pseudomonas aeruginosa, Acinetobacter baumannii, Klebsiella pneumoniae, and carbapenem-resistant *Enterobacteriaceae* (CRE) (5, 6). In addition, colistin has been extensively used in food animals, particularly in swine, since the 1960s for the control of *Enterobacteriaceae* infections and for growth promotion (7).

Coincident with increased use of colistin and polymyxin B, there have been increasing reports of resistance to these polymyxins. The most common mechanism for polymyxin resistance is via modifications of LPS on the outer membrane (5, 8, 9). Specifically, resistance occurs due to modification of the 1′ and/or 4′ phosphate groups of lipid A by cationic molecules to reduce the net negative charge of lipid A and block the binding of positively charged polymyxin. The modifications primarily consist of the addition of 4-amino-4-deoxy-l-arabinose (l-Ara4N) by l-Ara4N transferase (ArnT) and/or the addition of phosphoethanolamine (PEA) by PEA transferases (5, 8, 9). In Gram-negative bacteria, modification of lipid A by l-Ara4N and/or PEA is governed by the PmrA/PmrB and PhoP/PhoQ two-component regulatory systems, which are switched on by extracellular stimuli such as low pH, low cation (Ca^2+^ or Mg^2+^) concentrations, and the presence of cationic antimicrobial peptides (5, 8, 9). However, chromosomal mutations of genes in these systems can cause constitutive activation of the systems, resulting in modification of lipid A with l-Ara4N and pEtN groups under all growth conditions and leading to polymyxin resistance (10–12). These chromosomal mutations are an important source of resistance. For example, *mgrB* mutations are a frequent source of colistin resistance in Klebsiella pneumoniae (13).

In late 2015, a plasmid-mediated colistin resistance gene, *mcr-1*, was identified from an E. coli isolate from a pig farm in China (14). However, *mcr-1*-bearing plasmids have now been identified worldwide in animal, human, and environmental strains of *Enterobacteriaceae* (14, 15). In addition, *mcr-1* was also found in multidrug-resistant *Enterobacteriaceae* that contain extended-spectrum β-lactamase and carbapenemase genes (16, 17). This is of particular concern for the spread of pan-drug resistance genes in *Enterobacteriaceae*.

**FIG 2:**
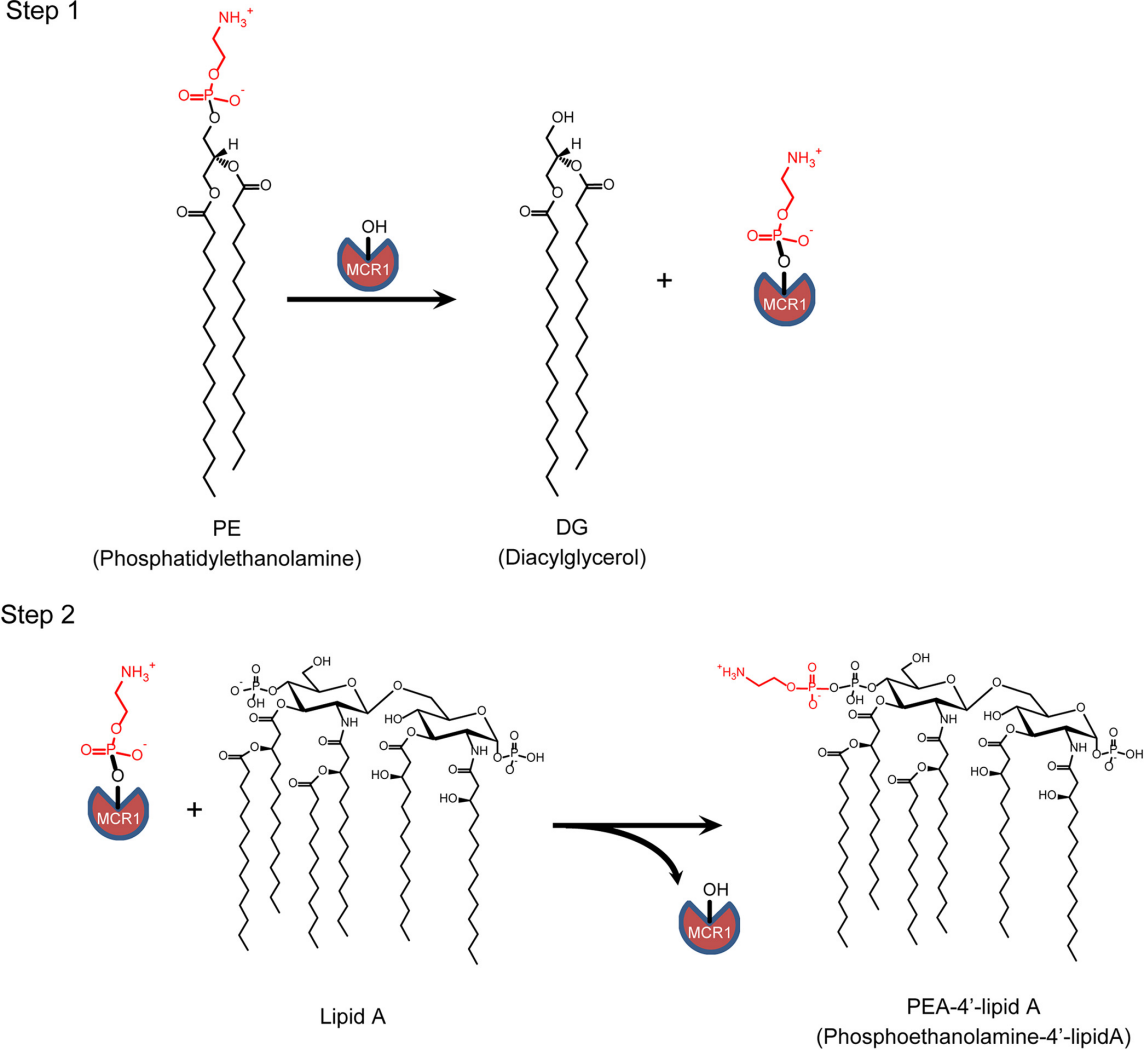
Phosphoethanolamine (PEA) transfer reaction catalyzed by MCR-1. The enzyme reaction is postulated to use a ping-pong mechanism that consists of two steps. In the first step, Thr285 of MCR-1 carries out a nucleophilic attack on the phosphate of the PEA moiety (red) of phosphatidylethanolamine (PE), resulting in the formation of a PEA–MCR-1 intermediate and the production of diacylglycerol (DG). In the second step, the 1′- or 4′-phosphate of lipid A acts as a nucleophile to attack the phosphate of the PEA-MCR-1 intermediate. PEA-lipid A is produced and MCR-1 enzyme is liberated. Although PEA modification is shown here to occur at the 4′ position of lipid A, it can occur at the 1′ position as well.

*mcr-1* encodes a membrane-localized PEA transferase, MCR-1, with a transmembrane domain at the N terminus and a soluble catalytic domain (cMCR-1) at the C terminus (18). Similar to other PEA transferases, MCR-1 is proposed to transfer the PEA group from phosphatidylethanolamine (PE) to the 1′- or 4′-phosphate group of lipid A (Fig. 2) (18). The enzyme reaction can be divided into two steps for which formation of a PEA-MCR-1 covalent intermediate is involved (18, 19). The first step of the enzyme reaction has been verified by *in vitro* experiments with purified MCR-1 protein and PE, while the second step is inferred from the production of PEA-modified lipid A in E. coli cells expressing MCR-1 protein (19–21).

We and others determined the X-ray crystal structure of the catalytic domain of the MCR-1 enzyme (cMCR-1), and a zinc ion is found to be coordinated at the putative active site of MCR-1 enzyme (18, 22–25). However, these structures revealed no obvious substrate binding sites in cMCR-1. This suggests the transmembrane domain of MCR-1 enzyme contributes to the formation of substrate binding sites in addition to localizing the enzyme to the cytoplasmic membrane of bacteria. The structure of full-length MCR-1 remains elusive to date. Therefore, to aid the understanding of mechanism of function of MCR-1 enzyme, we aimed to determine the active-site sequence requirements for the polymyxin resistance function of MCR-1. For this purpose, single-codon randomization libraries were constructed targeting 23 residues in and near the presumed active site of MCR-1 enzyme. The libraries were then selected to obtain functional mutants that supported the growth of E. coli cells in the presence of colistin or polymyxin B. Deep sequencing of the functional mutants revealed the frequency of occurrence of each amino acid at each randomized position before and after polymyxin selection. Wild-type amino acids were found to predominate in polymyxin-selected libraries for 17 residues positions, which include zinc-chelating residues, putative PE-interacting residues, and those involved in the formation of hydrogen bond networks in the active site. Amino acid substitutions at these residue positions significantly decreased polymyxin resistance and the PEA transferase activity of MCR-1. Therefore, they are essential for MCR-1 function and polymyxin resistance.

## RESULTS

### Transferase activity of MCR-1 enzyme is specific to PE as the donor substrate.

**FIG 3:**
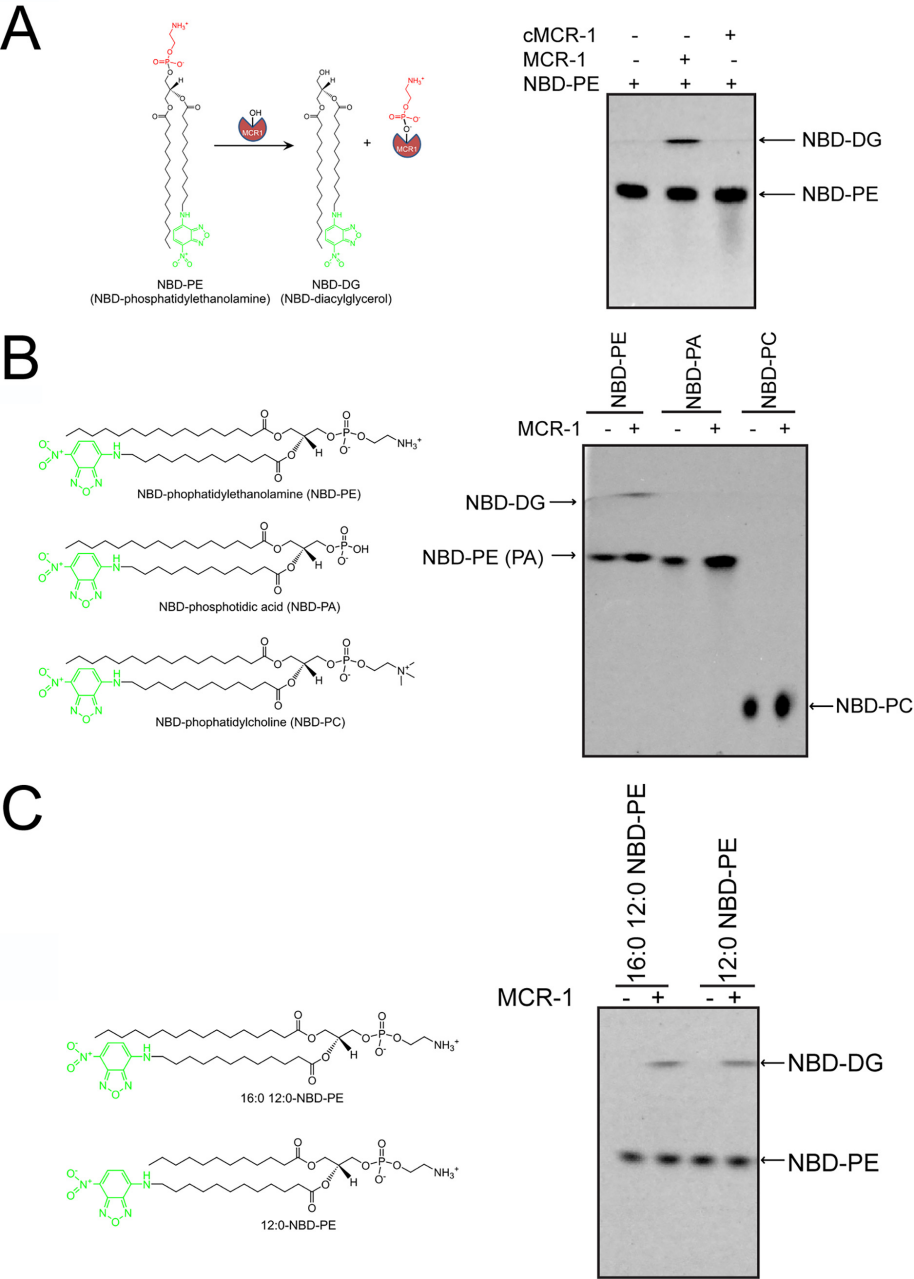
Enzyme activity of full-length MCR-1 and catalytic domain of MCR-1 (cMCR-1). (A) Enzyme activity of MCR-1 and cMCR-1 in converting NBD-phosphatidylethanolamine (NBD-PE) to NBD-diacylglycerol (NBD-DG). (B) Enzyme activity of MCR-1 in catalyzing different phospholipids. NBD-PA, NBD-phosphatidic acid; NBD-PC, NBD-phosphatidylcholine. (C) Enzyme activity of MCR-1 in catalyzing PE with different acyl chains. 16:0-12:0 NBD-PE, 1-palmitoyl-2-(12-NBD)-sn-glycero-3–phosphoethanolamine; 12:0 NBD-PE, 1-acyl-2-(12-NBD)-sn-glycero-3–phosphoethanolamine. Fluorescently labeled phospholipids (NBD-PE, -PC, and -PA) were incubated with MCR-1 or cMCR-1 in *n*-dodecyl-β-d-maltopyranoside (DDM) micelles. All of the reaction samples were analyzed by thin-layer chromatography (TLC) using ethyl acetate-methanol-water (7:2:1) as the developing solvent to differentiate product NBD-DG from the lipid substrate.

MCR-1 is a membrane protein with a 5-membrane span domain at the N terminus and a PEA transferase domain at the C terminus (14). To verify whether the N-terminal transmembrane domain is required for the enzyme activity of MCR-1, both full-length MCR-1 and the catalytic domain of MCR-1 (cMCR-1) were purified and tested for PEA transferase activity using fluorescently labeled phosphatidylethanolamine, 16:0-12:0 (7-nitro-2–1,3-benzoxadiazol-4-yl) amino dodecanoyl (NBD)-PE, as the substrate. The reaction samples were subsequently applied to thin-layer chromatography (TLC) plates to differentiate the product, NBD-DG, from the substrate. As shown in Fig. 3A, although clear production of NBD-DG was observed after NBD-PE was incubated with full-length MCR-1 protein, there was no production of NBD-DG when NBD-PE was incubated with cMCR-1. This indicates that the transmembrane domain is required for the PEA transferase activity of MCR-1.

The PEA transfer reaction catalyzed by MCR-1 has been proposed to start with nucleophilic attack at the phosphate of the PEA moiety of PE (Fig. 2) (19, 26). However, the substrate specificity of the MCR-1 enzyme has not been characterized. For example, phosphatidylcholine (PC), which is also a major component of cytoplasmic membrane, has a structure similar to that of PE, except that PC has a bulkier head group than PE, with three additional methyl groups (Fig. 3B). In addition, phosphatidic acid (PA) has a smaller head group than PE (Fig. 3B). Therefore, we tested whether the bulkiness of head groups of phospholipids affects the transferase activity of MCR-1 enzyme by incubating NBD-labeled PE, PC, or PA with the MCR-1 enzyme. The reaction samples were then applied to a TLC plate to test for the presence of NBD-DG. As shown in Fig. 3B, in contrast to the reactions with NBD-PE, incubation of MCR-1 enzyme with NBD-PC or NBD-PA did not produce any NBD-DG. These results suggest the substrate binding pocket of MCR-1 is tailored for the head group of PE. The increased size of the head group of NBD-PC may result in steric clash, while the decreased size of the head group of NDB-PA may not provide sufficient binding energy for the coordination of the lipid in the active site of the MCR-1 enzyme.

The acyl chains of PE also have been proposed to be involved in the binding of the lipid substrate to the MCR-1 enzyme (21). Therefore, we examined the impact of the size of acyl chains of PE on the transferase activity of MCR-1 enzyme. For this purpose, NBD-PE with 12 carbons on both acyl chains (12:0 NBD-PE) and NBD-PE with 12 carbons on one acyl chain and 16 carbons on the other chain (16:0-12:0 NBD-PE) were incubated with MCR-1 enzyme, and the production of NBD-DG was detected by TLC. As shown in Fig. 3C, NBD-DG was produced in both reactions. Therefore, MCR-1 does not absolutely require a 16-carbon acyl chain for transferase activity.

### Modeled structure of full-length MCR-1 protein reveals the composition of the active site of the enzyme.

Although the structure of cMCR-1 has been determined, there are no obvious substrate binding sites in the soluble catalytic domain (18). In addition, as shown above, cMCR-1 does not display phosphoethanolamine transferase activity (Fig. 3A). Therefore, the transmembrane domain is required for the enzyme activity of MCR-1, possibly by contributing to the formation of substrate binding sites. Although the structure of full-length MCR-1 is not available, the crystal structure of a PEA transferase with 36% sequence identity to MCR-1, Neisseria meningitidis EtpA (*Nm*EtpA), has been solved (27). Therefore, the structure of *Nm*EptA was used as a template to model the structure of MCR-1 using the Swiss-Model workspace.

**FIG 4:**
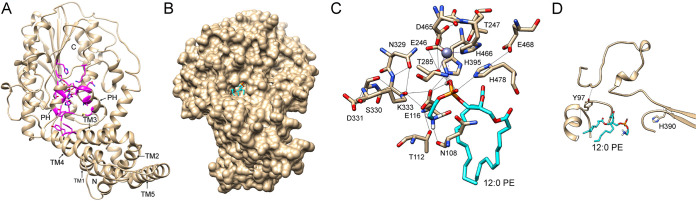
Molecular docking of phosphatidylethanolamine (PE) into the active site on the modeled structure of MCR-1 protein. (A) Ribbon diagram of the modeled structure of MCR-1. The positions of the five transmembrane segments (TMs) and two periplasmic helices (PHs) between TM3 and TM4 are indicated at the N terminus of the MCR-1 protein. The 23 active-site residues for which random libraries were created are shown in magenta. The zinc atom is represented as a gray sphere. (B) Surface representation of the docked 12:0 PE (cyan) into the putative substrate binding cavity of the MCR-1 enzyme. (C) Hydrogen bond network formed by the head group of PE and the active-site residues of MCR-1. The 12:0 PE molecule is shown in cyan. Hydrogen bonds are indicated by thin black lines. Zinc atom is represented as a gray sphere. (D) Hydrogen bonds involving residues Tyr97 and His390 of the MCR-1 enzyme. The 12:0 PE molecule is shown in cyan to indicate the substrate binding site. Hydrogen bonds are indicated by thin black lines.

As shown in Fig. 4A, the MCR-1 protein was modeled as two distinct domains, which are approximately perpendicular to each other. The N-terminal domain contains 5 transmembrane α-helices (TMs), which orient approximately parallel to each other. They are connected to the C-terminal catalytic domain (cMCR-1) via another α-helix and an extensive loop structure. Between TM3 and TM4 are two periplasm-facing helices (PHs). A surface representation of the modeled structure of MCR-1 protein shows that the two PHs, together with the end of TM3 and several residues on the C-terminal soluble domain, constitute a tunnel-shaped cavity (Fig. 4B). Two aromatic residues (Phe93 and Tyr97), three hydrophobic residues (Met105, Ala109, and Leu120), and one hydrophilic residue (Thr117) are located at the entrance of the tunnel; one hydrophobic residue (Leu477) and three polar residues (Asn108, Glu116, and Lys333) constitute the main body of the tunnel. The catalytic nucleophile residue Thr285, zinc ion, and other zinc-chelating residues (Glu246, Asp465, and His466) are located at the terminus of the tunnel. Although the model is based on a template with only 36% identity, the high sequence conservation of active-site residues among PE enzymes supports the placement of residues in the model. Furthermore, a 12:0 PE molecule can be docked into the cavity (Fig. 4B). Intriguingly, a similar cavity was also found on the structure of *Nm*EptA, and one molecule of DDM from the crystallization solution, which is similar to the substrate PE in structure, was found in the cavity (27). Amino acid sequence alignment between MCR-1 and *Nm*EptA reveals that the amino acid residues constituting the cavity are highly conserved between the two enzymes (27). Therefore, the tunnel-shaped cavity may represent the substrate binding and catalytic site of the MCR-1 and *Nm*EptA enzymes.

### Deep sequencing of polymyxin-selected randomization libraries of MCR-1 enzyme identifies active-site residues that are essential for its polymyxin resistance function.

To investigate the functional roles of those residues that constitute the tunnel-shaped cavity in the modeled MCR-1 structure, we targeted 23 residues around the cavity for construction of single-codon randomization libraries using site-directed mutagenesis with NNS degenerate primers, where N is any of the 4 nucleotides and S is C or G (Fig. 4A). The single-codon randomization libraries were used to select for mutants that provide polymyxin resistance by growing E. coli in broth culture medium containing colistin or polymyxin B. The cultures were then spread on nonselective plates to allow the formation of single colonies of functional MCR-1 mutants. The colonies from each experiment, i.e., naive (no selection) or polymyxin selection experiments, for each library were pooled, and plasmid DNA was prepared and used as the template for PCR to amplify the region of the *mcr-1* gene containing the randomized codon using primers with a 7-bp barcode sequence, which uniquely identifies each experiment. The barcoded PCR products, 69 in total (23 naive plus 23 colistin-selected plus 23 polymyxin B-selected), were purified from agarose gels, pooled, and subjected to paired-end next-generation sequencing (NGS).

The NGS sequence reads were assigned to each experiment based on the barcode sequences of the PCR amplicons and were computationally analyzed with custom scripts, which yielded 3.3 × 10^7^ total sequences for 69 experiments (Materials and Methods). This analysis entailed binning the sequences to each experiment, determining the nucleotide sequence of the relevant codon for each sequence read, and translating the codon into the encoded amino acid residue. The number of occurrences of each amino acid in each experiment was determined and shown in Fig. S1 and Table S1 in the supplemental material. In the naive libraries, each amino acid type had similar numbers of occurrences except for the libraries for residue positions Phe93, Gln111, Glu116, and Asn329, for which wild-type residues occurred more frequently than for any other amino acids (Table S1). The bias for wild-type residues at these positions is not clear but may be related to the fitness cost of amino acid substitutions at these residue positions for the growth of the library in E. coli.

10.1128/mBio.02776-21.1 FIG S1 Representative deep sequencing results of clones from naive and polymyxin-selected MCR-1 libraries. Download FIG S1, PDF file, 0.2 MB. Copyright © 2021 Sun and Palzkill. 2021 Sun and Palzkill. https://creativecommons.org/licenses/by/4.0/ This content is distributed under the terms of the Creative Commons Attribution 4.0 International license.

10.1128/mBio.02776-21.5 TABLE S1 Number of occurrences of each amino acid in MCR-1 randomization and selection experiment. Table S1, XLSX file, 0.02 MB Copyright © 2021 Sun and Palzkill. 2021 Sun and Palzkill. https://creativecommons.org/licenses/by/4.0/ This content is distributed under the terms of the Creative Commons Attribution 4.0 International license.

**FIG 5:**
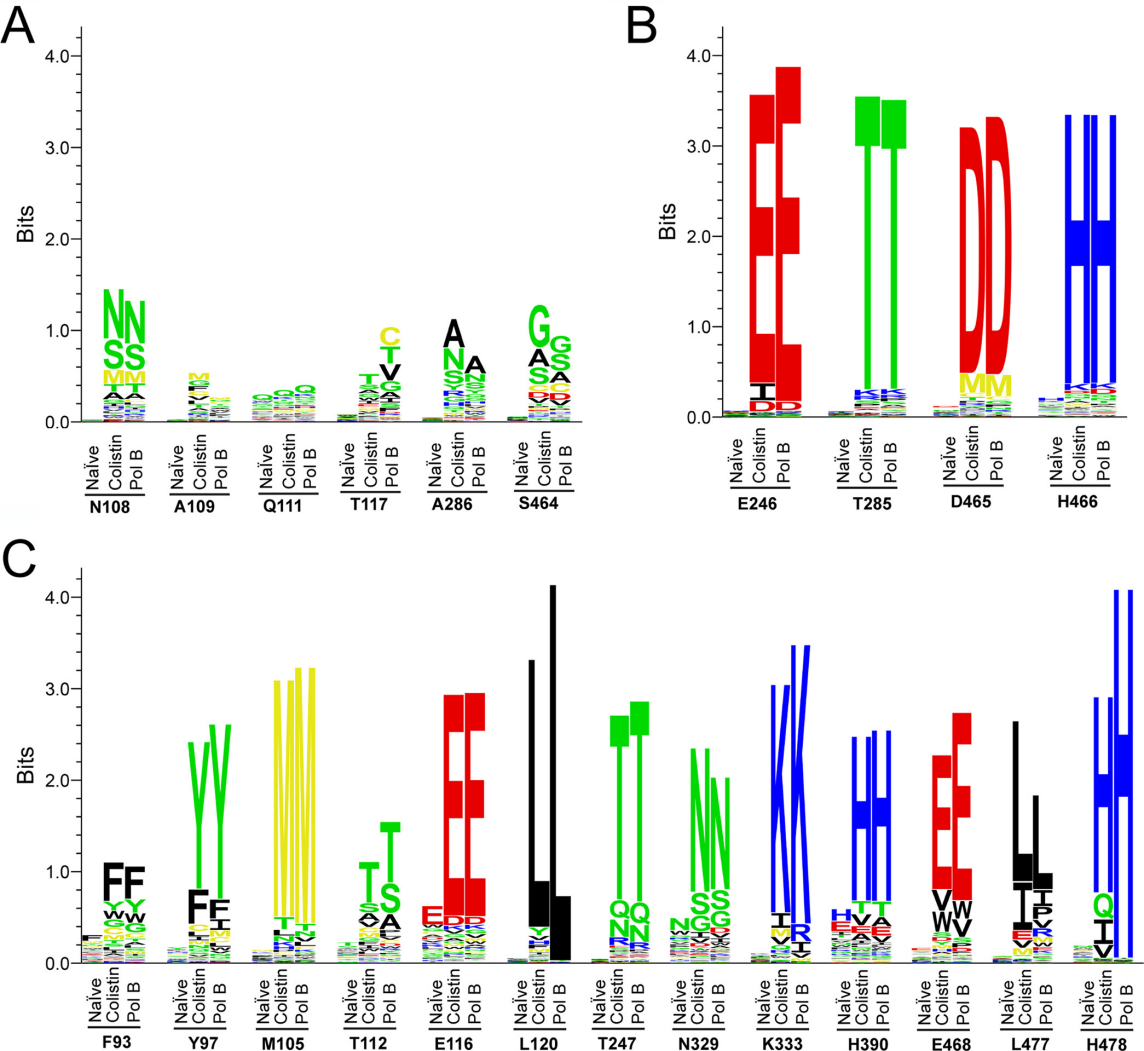
Sequence logos for nonessential and essential residues. (A) Nonessential residue class. For this class, multiple amino acid types occurred at a comparable frequency after selection of the libraries with either colistin or polymyxin B (Pol B), indicating relaxed sequence requirements at these positions. (B) Essential residue class, including the catalytic residue Thr285 and zinc-chelating residues. (C) Essential residue class not involved in chelating the zinc ion in the active site of MCR-1. For both essential residue classes, the wild-type residues dominate the sequences in the population after selection by either colistin or polymyxin B, indicating stringent sequence requirements at these positions.

As shown in Table S1, the selection for MCR-1-mediated polymyxin resistance resulted in the dominance of specific types of amino acids in the majority of the libraries. To visualize conservation of amino acids in each library after polymyxin selection, sequence logos were created based on the number of occurrences of each amino acid in each experiment (Fig. 5). The 23 active-site residues of MCR-1 can be placed into 2 groups based on whether there is dominance of specific amino acids after the polymyxin selections (Fig. 5). The first group included Asn108, Ala109, Gln111, Thr117, Ala286, and Ser464 (Fig. 5A). For these residue positions, neither the colistin nor polymyxin B antibiotic resistance selections resulted in the enrichment of specific amino acids in the library, indicating that many different amino acid types at these positions are consistent with MCR-1 function. The results suggest these positions do not contribute substantially to MCR-1 function. The second group of residue positions is defined as essential for function because the wild-type amino acid at these positions was highly enriched by the polymyxin selection and is dominant in the selected mutant population (Fig. 5B and C). Among these 17 residues, Thr285, which functions as the nucleophile in catalysis, was strongly enriched, as expected based on its established role in the reaction (18). In addition, the zinc-chelating residues Glu246, Asp465, and His466 were highly enriched by the polymyxin selections. These results are consistent with the requirement for a zinc ion to stabilize the activated Oγ of Thr285 for attack on the phosphate of the PE substrate (26).

To evaluate the contribution of the other active-site residues that were identified as essential based on sequencing results, the PEA donor substrate PE was docked into the active site of MCR-1 (Fig. 4B). In the docking model, the amine of PE forms hydrogen bonds with the side chain of Glu116, while the phosphate of PE forms hydrogen bonds with the side chains of Glu116, Thr285, His395, and His478 (Fig. 4C, Fig. S2). A hydrogen bond network also is formed surrounding the PEA moiety of PE by Thr112, Thr247, Asn329, Ser330, Lys333, and Glu468 together with PEA-interacting residues (Fig. 4C). In addition, Phe93, Met105, Leu120, and Leu477, which are located near the entrance of the tunnel-shaped cavity on the MCR-1 structural model (Fig. S2), form hydrophobic interactions with the acyl chains of PE to facilitate substrate binding to the cavity of MCR-1 (Fig. S2). Although Tyr97 and His390 do not interact directly with PE or catalytic residues of the MCR-1 enzyme, they may play an important role in maintaining structural integrity of the enzyme, as their main chains and/or side chains form hydrogen bonds with residues on the neighboring loops and strands (Fig. 4D). Therefore, these residues may play an important role in coordinating PE in the active site of MCR-1 and/or stabilizing the structure of MCR-1 and, therefore, are not tolerant of amino acid substitutions.

10.1128/mBio.02776-21.2 FIG S2 Ligand-protein interactions in the phosphatidylethanolamine (PE) docking model depicted using LIGPLOT. Download FIG S2, PDF file, 0.1 MB. Copyright © 2021 Sun and Palzkill. 2021 Sun and Palzkill. https://creativecommons.org/licenses/by/4.0/ This content is distributed under the terms of the Creative Commons Attribution 4.0 International license.

Several residues that were found to tolerate substitutions based on the sequencing results also appear to have interactions in the active site. In the docking model, the main-chain oxygen of Asn108 was found to form a hydrogen bond with the amine of the PEA moiety of PE, yet this residue could be replaced by other amino acids, including Ser and Met (Fig. 4C, Fig. S2). However, because the hydrogen bond is to the main chain of Asn108, these substitutions may retain positioning of the main chain for hydrogen bonding to PEA (Fig. 4C and 5A, Fig. S2). In addition, Ala109, Gln111, Thr117, and Ala286 were found to form hydrophobic interactions with PE (Fig. S2). However, these interactions may not contribute significantly to the binding of PE to the active site of MCR-1, as they can be freely substituted without interfering with the polymyxin resistance function of MCR-1 (Fig. 5A, Table S1). Ser464 is located in a loop structure adjacent to the zinc-chelating residue Asp465. Although Ser464 was not strongly enriched after polymyxin selection, amino acids with small side chains (Gly, Ala, and Ser) were predominant in the selected population (Fig. 5A, Table S1), indicating the importance of flexibility of the loop to the function of MCR-1.

In summary, deep sequencing of polymyxin-selected randomization libraries of MCR-1 identified 17 residue positions in and near the active site that are essential for the function of the MCR-1 enzyme. These results indicate the active-site residues are, in general, sensitive to substitutions, with 17/23 positions requiring the wild-type residue for function. In particular, the residues making interactions with the zinc or PE moiety or forming a hydrogen bond network exhibit stringent sequence requirements, while the residues making less specific hydrophobic interactions with the acyl chains of the substrate show more relaxed requirements.

### Relative fitness of MCR-1 mutants is consistent between colistin and polymyxin B selection experiments.

Although sequence logos in Fig. 5 reveal the conservation of amino acids in each library after polymyxin selection, they lack quantitative information on the change of polymyxin resistance function of each mutant. Therefore, the relative fitness ($F_{u}^{a}$) of each mutant in each experiment was calculated based on the frequency of occurrence of each amino acid in the naive and colistin- or polymyxin B-selected libraries, as described in Materials and Methods. The $F_{u}^{a}$ values of each mutant for colistin and polymyxin B were compiled into heat maps, as shown in Fig. S3A. Any amino acid substitutions at essential residues, such as Leu120, Glu246, Thr247, and Thr285, caused a significant decrease in fitness under both colistin and polymyxin E selection. On the contrary, multiple alleles exhibited $F_{u}^{a}$ values similar to those of the wild-type alleles for nonessential residue positions, including examples such as N108S, Q111K, and S464C, indicating that there is little or no change in polymyxin resistance function for the corresponding substitutions. Finally, a few substitutions result in enhanced fitness above wild-type levels, including S464A, A109F, and Q111K. The alleles with enhanced fitness occur exclusively at nonessential positions (Fig. S3A). Overall, however, examination of the heat map of fitness effects shows that substitutions that reduce fitness greatly outnumber those that are neutral or enhance activity.

10.1128/mBio.02776-21.3 FIG S3 Relative fitness of MCR-1 mutants under selection with colistin or polymyxin B. Download FIG S3, PDF file, 0.2 MB. Copyright © 2021 Sun and Palzkill. 2021 Sun and Palzkill. https://creativecommons.org/licenses/by/4.0/ This content is distributed under the terms of the Creative Commons Attribution 4.0 International license.

In addition, the $F_{u}^{a}$ values of each mutant were similar after selection of the libraries with either colistin or polymyxin B. To examine the correlation, the value of $F_{u}^{a}$ for each mutant from the colistin selection experiments was plotted versus the $F_{u}^{a}$ value of the same mutant from the polymyxin B selection experiments. As shown in Fig. S3B, linear regression analysis of the plot revealed a significant positive relationship with a slope of 1.07 between the fitness values for MCR-1 mutants selected by growth in either colistin or polymyxin B. This indicates that amino acid substitutions in MCR-1 result in a similar overall effect on colistin and polymyxin B antibiotic resistance levels and, presumably, on enzyme function. This result is consistent with the high degree of similarity in the chemical structures of colistin and polymyxin B (Fig. 1).

### Amino acid substitutions at essential residue positions abolish or significantly decrease polymyxin resistance levels conferred by MCR-1.

Deep sequencing of the MCR-1 libraries identified 17 active-site residues that are not tolerant and 6 residue positions that are tolerant of substitutions based on polymyxin antibiotic resistance selections. To validate the deep-sequencing results using a different method, amino acid substitutions were introduced at these residue positions and their effect on polymyxin resistance was tested. As shown in Table 1, E. coli cells expressing wild-type MCR-1 enzyme exhibited 32-fold higher resistance levels against both colistin and polymyxin B than control cells. Consistent with the deep-sequencing results, alanine substitutions at any of the 17 essential residues of MCR-1 resulted in 8- to 32-fold decreased resistance toward both colistin and polymyxin B. In addition, conservative substitutions at zinc-chelating residues of MCR-1 (E246D or D465E) caused a similar effect as alanine mutations on polymyxin resistance levels provided by the MCR-1 enzyme, again consistent with the sequencing results. In contrast, substitutions of residue positions classified as nonessential by deep sequencing by amino acids that occurred at similar frequency as the wild-type residue, such as N108S and S464G, did not alter either colistin or polymyxin B resistance levels compared to wild-type MCR-1. Therefore, MIC determinations for individual MCR-1 mutants are consistent with the conclusions from the deep-sequencing experiments. It is interesting that both wild-type MCR-1 and its mutants consistently showed higher resistance levels toward colistin than polymyxin B. This reflects the higher potency of polymyxin B than colistin in inhibiting both polymyxin-susceptible and -resistant cells, which may be due to altered hydrophobicity between the drugs resulting from the one amino acid difference in the cyclic heptapeptide ring, i.e., d-phenylalanine in polymyxin B versus d-leucine in colistin (Fig. 1A).

**TABLE 1:** Polymyxin resistance levels of E. coli cells expressing wild-type MCR-1-StrepII or mutants

MCR-1 WT/mutant	MIC (μg/ml)	
	Colistin	Polymyxin B
None	0.125	0.031
MCR-1 WT	4	1
M105A	0.25	0.0625
N108S	4	1
L120A	0.25	0.0625
E246D	2	0.5
E246A	0.125	0.031
T247A	0.25	0.125
T285A	0.25	0.0625
D465E	0.125	0.031
D465A	0.125	0.0625
E468A	0.5	0.125
S464G	4	1
H478A	0.25	0.0625

### Amino acid substitutions at essential residue positions decrease PEA transferase activity of MCR-1 protein.

Polymyxin resistance levels can be attributed to the enzyme activity of MCR-1 in transferring the PEA group from PE to lipid A. As shown in Fig. 2, MCR-1 catalyzes two consecutive nucleophilic reactions (18, 26). The first reaction of the Thr285 Oγ with the PE phosphate results in the production of diacyl glycerol (DG), while the second reaction of the lipid A phosphate on the Thr285 intermediate results in PEA modification of lipid A. This modification neutralizes negative charges on lipid A and, thus, decreases its affinity for the positively charged polymyxin, causing resistance against polymyxins (3).

**FIG 6:**
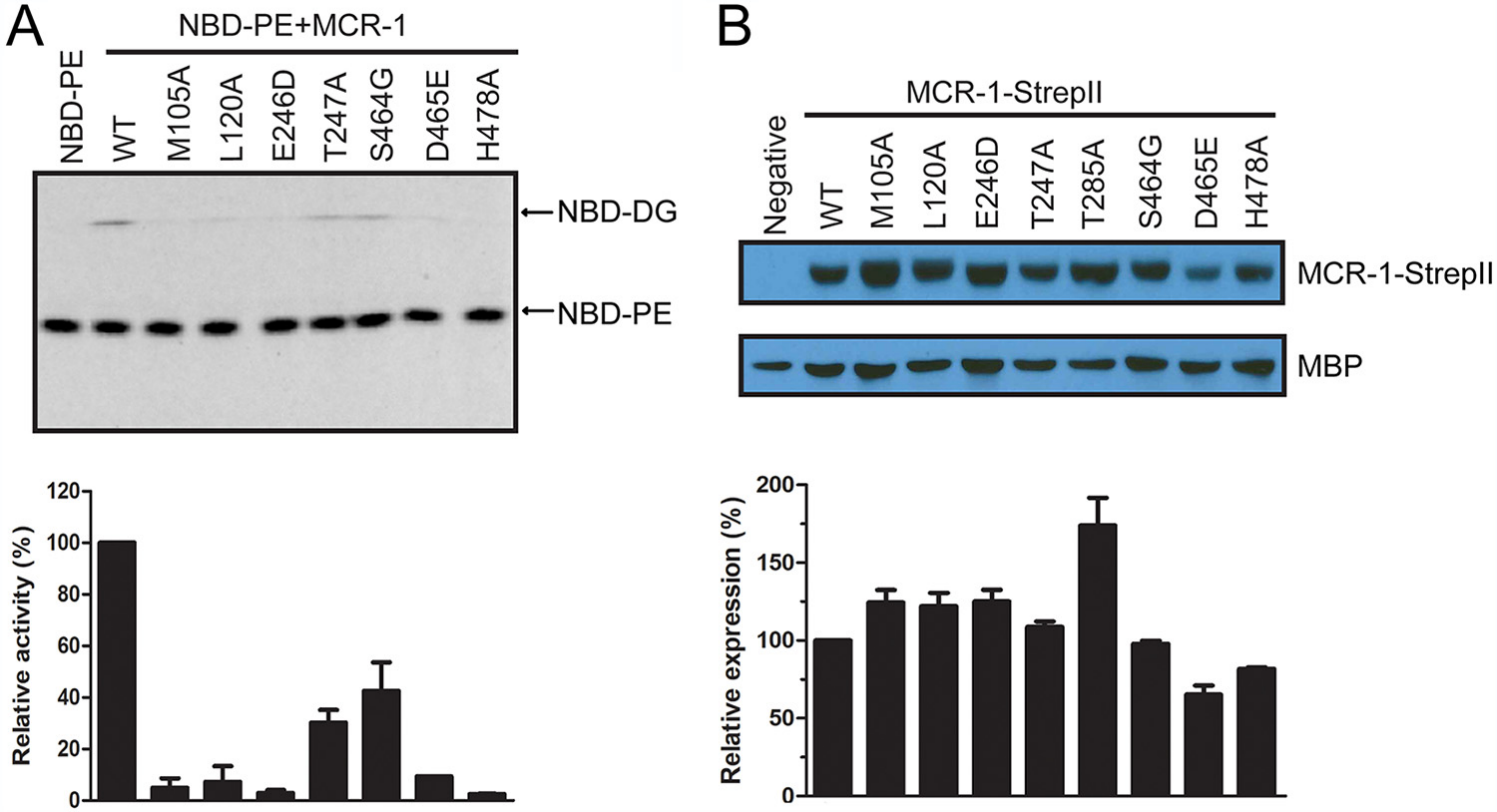
*In vitro* enzyme activity and *in vivo* steady-state protein levels of wild-type MCR-1 and mutants. (A) Enzyme activity of wild-type MCR-1 and mutants in converting NBD-phosphatidylethanolamine (NBD-PE) to NBD-diacylglycerol (NBD-DG). The samples were analyzed by thin-layer chromatography (TLC). The band for the product, NBD-DG, on the TLC plate was quantified by densitometry. The signal of NBD-DG for the reactions with mutant MCR-1 enzyme was normalized to that with the wild-type MCR-1 enzyme, which was set as 1. (B) *In vivo* steady-state protein levels of wild-type MCR-1 and mutants. Whole-cell lysates of mid-log-phase cultures of recombinant E. coli cells expressing wild-type or MCR-1 mutants were separated by SDS-PAGE, and protein levels of StrepII-tagged MCR-1 (MCR-1-StrepII) were determined by immunoblotting. The same membrane was also probed with polyclonal antibody specific for constitutively expressed maltose-binding protein (MBP), which is used as a loading control. The hybridization signal for wild-type and mutant MCR-1-StrepII and MBP was quantified by densitometry. The signal for MCR-1-StrepII was normalized to that for MBP in the same sample. Protein levels of mutant MCR-1-StrepII are expressed in the bar graph relative to that of the wild-type protein, which was set as 1. Data quantification is based on two independent experiments, and a representative result is shown.

To compare the enzyme activity of wild-type and mutant MCR-1 in catalyzing the first nucleophilic reaction, the production of DG in the enzyme reaction *in vitro* was quantified. For this purpose, fluorescently labeled PE (NBD-PE) was incubated with purified wild-type or mutant MCR-1 protein for half an hour at room temperature. After the enzyme reaction was stopped by the addition of cold methanol, 1 μl of reaction product was analyzed by TLC to differentiate fluorescently labeled product (NBD-DG) from NBD-PE. As shown in Fig. 6A, compared with wild-type MCR-1, mutation at the nonessential residue (S464G) only slightly decreased the production of NBD-DG. However, all tested mutations at essential residues except T247A markedly decreased the production of NBD-DG. This indicates that the tested essential residues except Thr247 are critical for MCR-1 in catalyzing nucleophilic attack on the phosphate of PE.

Besides enzyme activity, steady-state expression levels of MCR-1 protein also contribute to its polymyxin resistance phenotype. Therefore, the protein expression levels of wild-type and mutant MCR-1 were determined by immunoblotting using antibodies recognizing the StrepII tag at the terminus of the MCR-1 protein. As shown in Fig. 6B, with the exception of D465E, which decreased the steady-state protein level of MCR-1 by 25%, none of the tested mutations, including T247A, decreased the steady-state protein levels of MCR-1. Interestingly, alanine mutation at the nucleophile and zinc-chelating residue Thr285 caused an approximately 80% increase in the steady-state expression of MCR-1 protein, indicating that this mutation, although it blocks catalytic activity, stabilizes the MCR-1 protein. A similar effect of stabilizing an enzyme by mutating the catalytic nucleophile to alanine or glycine has been observed for serine active-site β-lactamases and may be related to reducing steric strain on the active site (28).

## DISCUSSION

Polymyxins, including colistin and polymyxin B, exert bactericidal activity by permeabilizing membranes of Gram-negative bacteria after binding to the negatively charged lipid A of LPS in the outer and inner membrane through electrostatic interaction (2, 3). Some Gram-negative bacteria are naturally resistant to polymyxins, while others can acquire polymyxin resistance via chromosomal mutations, which results in modification of lipid A with cationic molecules to neutralize its negative charge and block the binding of positively charged polymyxins (29). Addition of l-Ara4N and/or PEA to 1′- and/or 4′-phosphate groups of lipid A are the primary modes of lipid A modifications, and they are catalyzed by l-Ara4N transferase (ArnT) and PEA transferases, respectively (29).

The X-ray structure of the PEA transferase from Neisseria meningitidis (*Nm*EptA) has been determined, which consists of 5 transmembrane segments at the N terminus and a PEA transferase domain at the C terminus (27). In addition, a substrate binding cavity constituted by amino acids from both transmembrane segments and the PEA transferase domain was found on the structure of *Nm*EptA. One molecule of DDM from the crystallization condition, which is similar to the substrate PE in structure, was found to be coordinated in the binding cavity, indicating that PE, the donor substrate of the transferase, binds in the binding cavity. However, no other cavity was found on the structure of *Nm*EptA that can fit lipid A, the receiver substrate of the enzyme. Molecular dynamics simulations and intrinsic fluorescence studies indicate that *Nm*EptA undergoes conformational changes, possibly to accommodate the two lipid substrates with very different sizes (Fig. 2). Therefore, *Nm*EptA may use a ping-pong mechanism, which involves the formation of a PEA-enzyme intermediate, to catalyze the transfer of PEA group from PE to lipid A.

MCR-1 is a PEA transferase encoded by a plasmid-mediated colistin resistance gene, *mcr-1*, that was recently identified in E. coli and other Gram-negative species from various origins (14, 15). Analysis of the MCR-1 protein sequence revealed that MCR-1 has 36% sequence identity to *Nm*EptA and, similar to *Nm*EptA, may form a 5-helix transmembrane domain at the N terminus and a soluble catalytic domain at the C terminus (14, 18). The crystal structure of the catalytic domain of MCR-1 (cMCR-1) has been determined by several groups (18, 22–25). However, the substrate binding site is not obvious on the structure of cMCR-1. This may account for the failure of cMCR-1 to catalyze the conversion of PE to DG (Fig. 3A) and indicates the requirement of the transmembrane domain for PEA transferase activity of MCR-1 enzyme.

Previous work with *Nm*EptA enzyme showed that the PEA transferase is promiscuous with regard to the alkyl chain identity of the donor substrate PE as the enzyme, when encapsulated within a nanostructured bicontinuous cubic phase, transfers the PEA moiety from PE with unsaturated acyl chains (1,2-dioleoyl-glycero-phosphoethanolamine, or DOPE; 18:1-PE) as efficiently as that with saturated acyl chains (1,2-dimystroyl-glycero-phosphoethanolamine, DMPE; 14:0-PE) to the receiver substrate (30). Here, we also find that the length of the acyl chain of PE has little, if any, effect on the enzyme activity of MCR-1 (Fig. 3C). In contrast, the identity of the head group of the donor substrate is crucial to the enzyme activity of MCR-1, as it can only catalyze the conversion of PE to DG but not that of PA or PC, which differ from PE only in the head group (Fig. 3B). Therefore, MCR-1 transferase activity is specific to the PE as the donor substrate. Furthermore, in the work with *Nm*EptA enzyme, it was found that *Nm*EptA could transfer the PEA moiety from PE to monoolein to form monoolein phosphoethanolamine (MOPE) (30). Considering that monoolein, which is a monoacylglycerol, is structurally distinct from lipid A by both acyl chain tail and head groups, *Nm*EptA may have no specificity on the PEA receiver substrate (30). Therefore, determining whether MCR-1 can transfer the PEA group from PE to lipidic substrates besides lipid A is worthy of further investigation.

Unlike its homologue, *Nm*EptA, the structure of full-length MCR-1 has not been determined. Therefore, we generated a modeled structure of MCR-1 using the structure of *Nm*EptA as a template. As in the *Nm*EptA enzyme, two distinct domains, i.e., the transmembrane domain and catalytic domain, are apparent in the modeled structure (Fig. 4A). The active site of MCR-1 is located at the interface of the two domains in a tunnel-shaped cavity (Fig. 4B). The PE substrate can be docked into the cavity structure (Fig. 4B).

**FIG 7:**
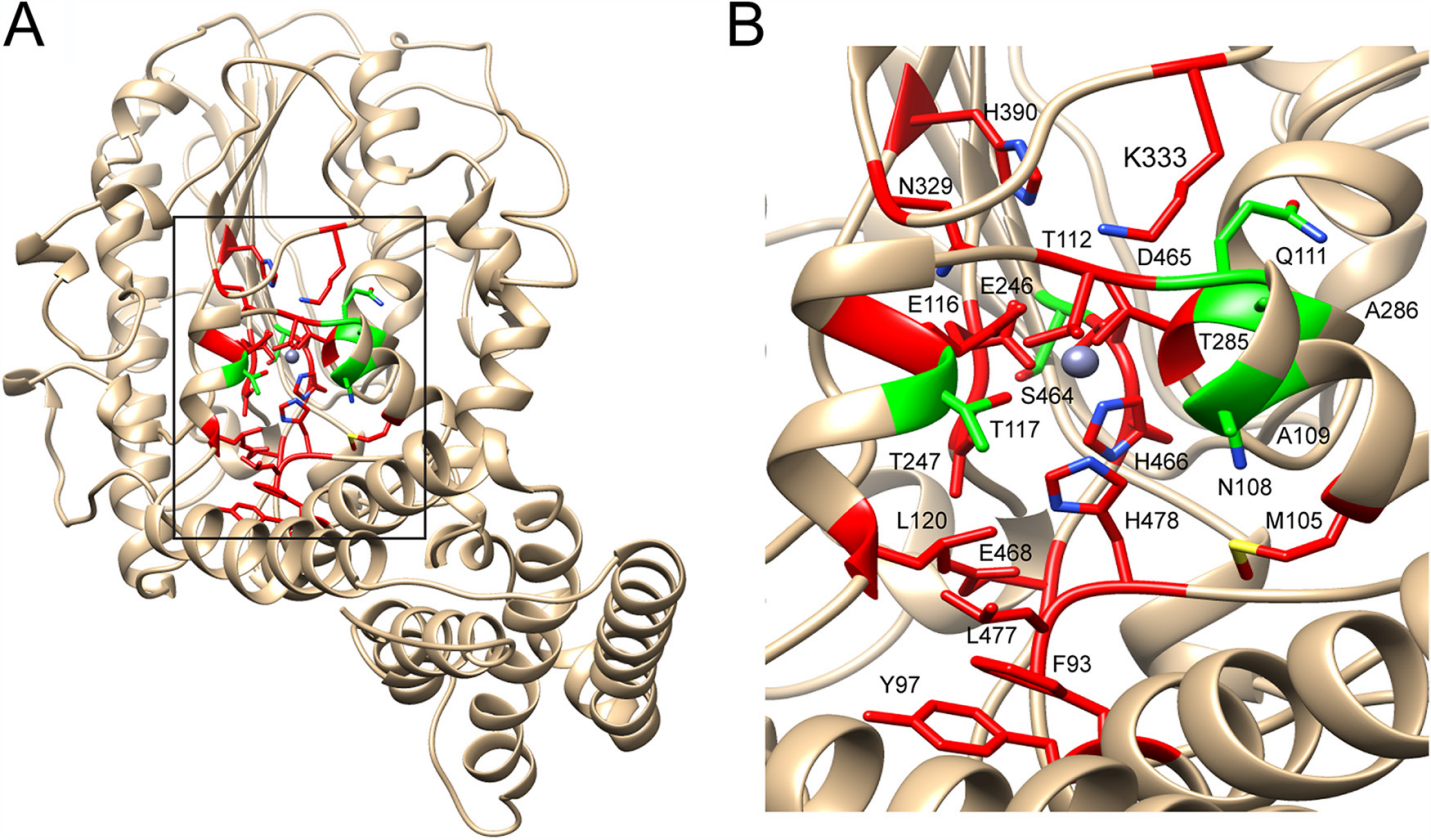
Ribbon diagram of the modeled structure of the MCR-1 enzyme showing the location of essential and nonessential residues. Essential and nonessential residues are labeled red and green, respectively, whereas the zinc ion is represented as a gray sphere. The box in panel A indicates the region of the structure shown in panel B.

To understand the functional importance of amino acid residues that constitute the tunnel-shaped cavity, we individually randomized these positions by constructing single-codon randomization libraries. After selection of the libraries for supporting cell growth in the presence of colistin or polymyxin B, the functional mutants were subjected to deep sequencing to identify the frequency of occurrence of each amino acid at each residue position (see Table S1 in the supplemental material). The sequencing results revealed that a wide distribution of amino acid types is observed after selection of the libraries for 6 residue positions (Fig. 5A), suggesting that they are nonessential for polymyxin resistance function of MCR-1 enzyme. However, for the libraries of the other 17 residue positions, wild-type amino acids were found to be conserved after selection by either colistin or polymyxin B (Fig. 5B and C), indicating that they are essential for the polymyxin resistance function of MCR-1 protein. Both essential residues and nonessential residues are shown mapped on the modeled structure of MCR-1 in Fig. 7. It appears that side chains of most nonessential residues orient away from the active site of MCR-1, suggesting they play a minor role, if any, in binding substrates of MCR-1 enzyme.

Essential residues include the nucleophile Thr285 and other zinc-chelating residues (Asp246, Asp465, and His466). Docking of PE into the active site of MCR-1 showed that other essential residues form a hydrogen bond network together with the PEA moiety of PE (Thr112, Glu116, Thr247, Asn329, Lys333, Glu468, and His478) or form hydrophobic interactions with the acyl chains of PE (Phe93, M105, L120, and Leu477) (Fig. 4C, Fig. S2). These residues may be critical for enzyme activity by stabilizing the alkoxide of the nucleophile Thr285 and/or coordinating the PE substrate in the active site so that Thr285 can carry out an efficient nucleophilic attack on the phosphate group of PE, or, in the case of the hydrophobic interactions, facilitate substrate binding. Therefore, any amino acid substitutions at these residues significantly decrease the PEA transferase activity and polymyxin resistance function of MCR-1 (Fig. 6A and Table 1) (21). However, it is interesting that the T247A substitution only decreased the enzyme activity of MCR-1 in converting PE by 40% but abolished polymyxin resistance (Fig. 6A and Table 1). This suggests that Thr247 is more important for the second step of the PEA transfer reaction, i.e., transfer of PEA from the MCR-1–PEA intermediate to the phosphate group of lipid A (Fig. 2). Since an *in vitro* assay to demonstrate the activity of MCR-1 in catalyzing the second step of the reaction is lacking, molecular docking was attempted to place lipid A in the active site to illustrate how lipid A is coordinated in MCR-1. However, docking was not successful even when the acyl chains of lipid A were totally removed. This indicates that a bigger cavity is required for the binding of bulky lipid A to the active site of MCR-1, which can be achieved via conformational change of MCR-1 protein during catalysis, as visualized for the structure of *Nm*EptA (27).

Since the report of MCR-1 in late 2015, 10 MCR homologues have been identified, mainly in *Enterobacteriaceae* (14, 31–39). A multiple-sequence alignment shows that they only share 16% sequence identity, and their transmembrane domains are more variable than catalytic domains (Fig. S4). However, all of the essential residues identified in this study except Phe93, Leu120, and Asn329 are conserved in the 10 MCR homologues. In addition, 13 out of the 17 essential residues are also conserved in MCR homologues and chromosomal PEA transferases from E. coli (*Ec*EptA) and N. meningitidis (*Nm*EptA). This suggests that although mobile and chromosomal PEA transferases have varied protein sequences, they utilize an evolutionarily conserved mechanism to modify lipid A. This is supported to some extent by the finding that the transmembrane domains of MCR-1 and MCR-2 enzymes can be exchanged without losing their PEA transferase activity (19). Therefore, because the residues that are essential are also conserved, we suggest that development of inhibitors that target the active-site residues shown here to be essential for function have the potential to inhibit most, if not all, homologues of the MCR enzymes and thereby circumvent polymyxin resistance caused by MCR.

10.1128/mBio.02776-21.4 FIG S4 Multiple sequence alignment among 10 MCR homologues and chromosomal phosphoethanolamine transferases. Download FIG S4, PDF file, 0.3 MB. Copyright © 2021 Sun and Palzkill. 2021 Sun and Palzkill. https://creativecommons.org/licenses/by/4.0/ This content is distributed under the terms of the Creative Commons Attribution 4.0 International license.

## MATERIALS AND METHODS

### Bacterial strains and expression vectors.

E. coli XL1-Blue (Stratagene) and E. coli BL21(DE3) were used as the host strains for the construction of codon randomization libraries and for overproduction of wild-type or mutant MCR-1 enzyme, respectively. For the construction of MCR-1 randomization libraries, a Strep tag II-fused MCR-1 (MCR-1-StrepII) was amplified from pBCKSII-MCR-1 (18) using *Pfu* polymerase (Invitrogen) and cloned between SacI and XbaI restriction sites of the chloramphenicol-resistant pTP470 plasmid to obtain MCR-1-StrepII-pTP470, on which the expression of MCR-1 was under the control of an isopropyl-β-d-thiogalactopyranoside (IPTG)-inducible *trc* promoter. The inclusion of Strep tag II at the C terminus of MCR-1 was used to monitor the expression of MCR-1 in E. coli by immunoblotting with anti-Strep tag II antibody. The Strep tag II-modified MCR-1 plasmid construct was shown to provide colistin resistance to E. coli XL1-Blue cells.

For overexpression and purification of MCR-1, *mcr-1* was cloned between NdeI and XhoI restriction sites of a modified pET28a vector (Novogen), in which the thrombin recognition sequence was replaced with the tobacco etch virus (TEV) protease recognition sequence and 2 extra codons for histidine residues were inserted before the TEV protease recognition sequence to increase binding affinity of His-tagged target proteins to the HisTrap columns. Site-directed mutagenesis was performed on MCR-1-StrepII-pTP470 and MCR-1-pET28a-TEV to obtain expression vectors for MCR-1-StrepII and His-MCR-1 mutants.

### Molecular modeling of full-length MCR-1 structure and docking of PE onto the active site of the modeled structure of full-length MCR-1.

Although the structure of full-length MCR-1 has not been solved, the crystal structure of a homologue of MCR-1 with 36% sequence identity, Neisseria meningitidis phosphoethanolamine transferase (*Nm*EptA; PDB entry 5FGN), has been determined (27). Therefore, the structure of MCR-1 was modeled on the Swiss-Model workspace (https://swissmodel.expasy.org/interactive) using the structure of *Nm*EptA as the template.

To reveal the binding mode of two lipid substrates in the active site of the MCR-1 enzyme, molecular docking was performed by using built-in AutoDock Vina in UCSF Chimera. First, a PDB file for 1,2-dipalmitoyl-sn-glycero-3-phosphoethanolamine (16:0 PE) was created by using Chimera with Canonical SMILES [CCCCCCCCCCCCCCCC(=O)OCC(COP(=O)(O)OCCN)OC(=O)CCCCCCCCCCCCCCC] from PubChem (https://pubchem.ncbi.nlm.nih.gov/). Hydrogens and charges were added to the PDB files of both 16:0 PE and MCR-1 to prepare for the docking. We attempted to dock 16:0 PE into the active site of MCR-1 using built-in AutoDock Vina in Chimera. However, possibly due to the length and flexibility of its acyl chains, 16:0 PE could not be docked into the active site of MCR-1, as reported by Xu et al. (21). Therefore, the acyl chains of 16:0 PE were shortened to 12 carbons to obtain 12:0 PE, which was successfully docked into the active site of the MCR-1 enzyme by using a grid size of 28 Å by 25 Å by 40 Å. Although multiple conformations of 12:0 PE were modeled in the active site of MCR-1 protein, only one of them was accepted based on highest binding energy and shortest distance between the phosphorus head group of 12:0 PE and hydroxyl group of Thr285, which is the nucleophile conserved in all known PEA transferases. LigPlot+ was then used to generate the two-dimensional interaction diagrams between 12:0 PE and MCR-1.

We also attempted to dock lipid A into the active site of MCR-1 using a strategy similar to that for PE. However, this was not successful, even after all of the acyl chains were removed from lipid A.

### Construction of single-codon randomization libraries of MCR-1.

Based on the modeled structure of the MCR-1 protein, single-codon randomization libraries targeting 23 residues in and near the active site of MCR-1 were constructed using site-directed mutagenesis as described previously (40). An XhoI restriction site was first inserted near the target codon to eliminate any wild-type MCR-1 background, as the insertion was designed to introduce a frameshift mutation in the MCR-1 gene. The XhoI insert mutant was then used as the template for randomization of each codon, which was replaced by NNS (where N is any of the 4 nucleotides and S is G or C) to represent all 20 amino acids. The resulting mutagenesis reactions were treated with DpnI and XhoI before being transformed into E. coli XL1-Blue competent cells. More than 300 colonies were pooled for each library construction, and plasmid DNA was extracted to obtain a single-codon randomization library.

### Selection of MCR-1 libraries with resistance to colistin and polymyxin B.

The genetic selection of E. coli containing the MCR-1 libraries for colistin and polymyxin B resistance was carried out in E. coli XL1-Blue cells grown in 2× YT medium containing 12.5 μg/ml chloramphenicol using a method adapted from that developed by Stiffler et al. (41). We first tested the resistance levels of E. coli encoding wild-type MCR-1 for growth with various levels of colistin and polymyxin B. For that purpose, 10 ng MCR-1-StrepII-pTP470 plasmid was transformed into the E. coli XL1-Blue competent cells. After recovery, the transformed cells were grown overnight at 37°C with shaking in 2× YT medium containing 12.5 μg/ml chloramphenicol. The next morning, the overnight cultures were 1:500 diluted in 2× YT medium containing 12.5 μg/ml chloramphenicol and incubated at 37°C with shaking until the optical density at 600 nm (OD_600_) reached 0.01, and then 0.96 ml of the bacterial cultures was transferred to each well of a 96-well deep well plate containing 0.04 ml of increasing concentrations of polymyxin (0.8, 1.6, 3.2, 6.4, 12.8, or 25.6 μg/ml final concentration for colistin and 0.2, 0.4, 0.6, 0.8, 1.6, or 3.2 μg/ml polymyxin B). Meanwhile, bacterial cultures were also transferred to one well without any polymyxin, which was used as a growth control. The plate was then incubated at 37°C with shaking until the OD_600_ of the control cultures reached between 0.1 and 0.2. Finally, 0.1 ml of culture from each selection was spread onto 2× YT agar plates containing 12.5 μg/ml chloramphenicol, which were incubated overnight at 37°C to allow colony formation. The highest concentrations of polymyxin that allow the formation of more than 500 colonies were regarded as the polymyxin resistance level of E. coli encoding MCR-1-StrepII, which was 12.8 μg/ml for colistin and 1.6 μg/ml for polymyxin B.

For the selection of MCR-1 randomization libraries for polymyxin resistance, 50 ng library plasmid DNA was transformed into the E. coli XL1-Blue competent cells. The selection procedure was the same as that for testing MCR-1-StrepII, except a no polymyxin control (naive), 12.8 μg/ml colistin, and 1.6 μg/ml polymyxin B were included during the process. Finally, appropriate volumes of the cultures were spread onto the 2× YT agar plates containing 12.5 μg/ml chloramphenicol so that approximately 500 colonies were formed on the selective agar plates.

### Preparation of samples for Illumina sequencing.

Samples for Illumina sequencing were prepared as described previously (40, 42). Briefly, for each library, colonies from naive, colistin selection, or polymyxin B selection experiments were pooled. Plasmid DNA was then prepared for each selection experiment and used as the template for PCR with primers containing 7-bp barcode sequences that are unique to each experiment to amplify a 150-bp DNA fragment that covers the library region. After agarose gel purification, the PCR products from all 69 experiments (23 libraries times 3 experiments/library) were pooled in a single tube, which was processed for Illumina paired-end HiSeq sequencing (2 × 150-bp read length) (Novogene, Inc.).

### Analysis of deep Illumina sequencing results.

Illumina HiSeq sequencing returned 2 FASTQ files containing the sequencing reads and quality information. Data quality control report showed that read quality score was higher than 30 throughout the sequence. The sequencing results then were analyzed to obtain the occurrence of each amino acid type in each experiment using custom scripts as described previously (40, 42). The analyzed results for all of the 69 experiments are shown in Table S1 in the supplemental material.

### Creation of sequence logos.

From the results for the libraries after selection by colistin or polymyxin B, sequence logos were created based on the frequency of occurrence of each amino acid in both naive and polymyxin-selected experiments for each library as described previously (40, 42).

### Calculation of relative fitness.

The relationship between the deep sequencing results of each library for the colistin selection experiments and polymyxin B selection experiments was examined by determining the relative fitness ($F_{u}^{a}$), which refers to the fitness of the amino acid substitution *a* at each position *u*. It was calculated as described by Stiffler et al. (41) using the equation below:

$$F_{u}^{a}=\log_{10}\left[\frac{N_{u}^{\text{a,sel}}}{N_{u}^{a,\text{naive}}}\right]\;-\;\log_{10}\left[\frac{N_{u}^{\text{wt,sel}}}{N_{u}^{\text{wt,naive}}}\right]$$

where $N_{u}^{a,\text{ sel}}$ and $N_{u}^{a,\text{ naïve}}$ represent the mutant allele count in the selected and unselected (naive) residue position library and $N_{u}^{\text{wt, sel}}$ and $N_{u}^{\text{wt, naïve}}$ represent the wild-type allele count in the selected and naive residue position library, respectively.

### Determination of polymyxin resistance levels.

Polymyxin resistance levels of E. coli XL-1 Blue cells expressing MCR-1 wild-type and mutant enzymes were measured by determining MICs of colistin and polymyxin B in liquid broth culture using 2-fold serial dilutions of the polymyxins as described previously (42). Although the expression of MCR-1 protein is under the control of an IPTG-inducible *trc* promoter, the basal expression of MCR-1 in the absence of IPTG conferred 32-fold higher levels of resistance against both colistin and polymyxin than the control vector. In addition, the inclusion of 0.2 mM IPTG in the culture medium resulted in only a 2-fold further increase in polymyxin resistance levels for E. coli XL-1 Blue cells expressing wild-type MCR-1 protein (data not shown). Therefore, IPTG was not included in MIC determinations to reduce the complexity of the experiment.

### Determination of protein expression levels.

The effect of amino acid substitutions on steady-state expression levels of MCR-1-StrepII protein in E. coli was determined by Western blot hybridization using a horseradish peroxidase (HRP)-conjugated mouse monoclonal anti-Strep II antibody (Novogen) as described previously (40, 42). In addition, the expression of endogenous maltose binding protein (MBP) was also detected using MBP-specific antibody to serve as a loading control. The hybridization signals for both MCR-1-StrepII and MBP were quantified by densitometry using ImageJ software (NIH). The signal for MCR-1-StrepII was normalized to that for MBP in the same sample. The normalized signal for MCR-1 mutants was finally expressed relative to that for MCR-1 wild type, which was set as 1.

### Overexpression and purification of wild-type and mutant MCR-1 enzymes.

The MCR-1-pET28a-TEV plasmid was used for overexpression of 8× His-tagged-MCR-1 (His-MCR-1) enzyme in E. coli BL21(DE3) cells. The E. coli cells containing the MCR-1 expression plasmid were grown at 37°C to an OD_600_ of 0.8 in LB medium containing 25 μg/ml kanamycin. Expression of the His-MCR-1 protein was induced with 0.5 mM IPTG at 18°C for 16 h. The cells were pelleted and suspended in lysis buffer (20 mM HEPES, 150 mM NaCl, pH 7.4) and lysed using a French press and a short period of sonication. The cell debris was removed by centrifugation at 12,000 × *g* for 30 min, and the membranes were isolated by ultracentrifugation at 170,000 × *g* for 1.5 h at 4°C. His-MCR-1 was solubilized from the membranes by incubating the isolated membranes at 4°C overnight with gentle shaking with 20 mM *n*-dodecyl-β-d-maltopyranoside (DDM) in 20 mM HEPES, pH 7.4, 300 mM NaCl, 1× EDTA-free protease inhibitor cocktail (Gendepot). The solubilized His-MCR-1 was separated from the membrane debris by ultracentrifugation at 170,000 × *g* for 1 h at 4°C.

Solubilized His-MCR-1 was purified by metal-chelating chromatography by loading the supernatant from the ultracentrifugation onto a 1-ml HisTrap FF column (GE Healthcare), which had been equilibrated by buffer A (20 mM HEPES, pH 7.4, 300 mM NaCl, 20 mM imidazole, 0.5 mM DDM, 1 mM phenylmethylsulfonyl fluoride [PMSF]). After washing with 5 column volumes (CVs) of buffer A, the bound proteins were eluted with 0 to 100% buffer B (20 mM HEPES, pH 7.4, 300 mM NaCl, 500 mM imidazole, 0.5 mM DDM, 1 mM PMSF) over 10 CVs. Fractions containing His-MCR-1 were pooled, concentrated, and buffer exchanged to buffer A with a 50-kDa cutoff Amicon Ultra-15 centrifugal filter unit (EMD Millipore). The His tag was cleaved by incubating with TEV protease for 16 h at 4°C at a ratio of 1:50, and the TEV protease was removed by incubating with Ni Sepharose 6 fast-flow resin (GE Healthcare). MCR-1 was further purified by gel filtration chromatography using a Superdex 200 Increase GL 10/300 sizing column (GE Healthcare) with 20 mM HEPES, pH 7.4, 150 mM NaCl, 0.5 mM DDM, 1 mM PMSF as running buffer. Fractions containing MCR-1 were pooled and concentrated. The final protein concentration of MCR-1 was determined by measuring absorbance at 280 nm with a DU800 spectrophotometer (Beckman Coulter) and using an extinction coefficient of ε_280_ = 66,240 M^−1 ^cm^−1^, which was calculated using the ExPASy ProtParam tool. MCR-1 mutants were overexpressed, purified, and quantified in the same way as that for wild-type MCR-1 protein. SDS-PAGE analysis revealed that the purity of wild-type MCR-1 and each of the mutant enzymes was higher than 95%.

### Determination of enzyme activity of MCR-1 wild-type and mutant enzymes using NBD-labeled glycerophospolipid.

To determine glycerophospolipid substrate specificity of MCR-1 enzyme, 0.2 mM 1-acyl-2-(12-NBD)-sn-glycero-3-phosphoethanolamine (12:0 NBD-PE) (Avanti Lipids), 1-palmitoyl-2-(12-NBD)-sn-glycero-3-phosphoethanolamine (16:0-12:0 NBD-PE) (Avanti Lipids), 1-palmitoyl-2-(12-NBD)-sn-glycero-3-phosphocholine (16:0-12:0 NBD-PC) (Avanti Lipids), or 1-palmitoyl-2-(12-NBD)-sn-glycero-3-phosphate (16:0-12:0 NBD-PA) (Avanti Lipids) was incubated with 33 μM MCR-1 protein in the enzyme reaction buffer (50 mM HEPES, pH 7.4, 100 mM NaCl, 0.5 mM DDM) at room temperature for 16 h. The reaction products were diluted 1:100 with methanol, applied to a thin-layer chromatography (TLC) (Millipore) plate, and developed using ethyl acetate-methanol-water (7:2:1) (27). The fluorescence signal on the TLC plate was visualized in the Chemidoc MP imaging system (Bio-Rad) using the settings for Alx488 blot (excitation wavelength, 488 nM; emission wavelength, 532 ± 28 nm).

The phosphoethanolamine transferase activity of full-length MCR-1 and cMCR-1 was examined and compared using 0.2 mM 16:0-12:0 NBD-PE incubated with 33 μM full-length MCR-1 or cMCR-1 in the enzyme reaction buffer for 16 h. The reactions products were diluted 1:100 by methanol and analyzed by TLC.

To compare the phosphoethanolamine transferase activity between wild-type and mutant MCR-1 enzymes, 0.2 mM 16:0-12:0 NBD-PE was incubated with 3.3 μM wild-type or mutant MCR-1 protein in the enzyme reaction buffer at room temperature for 30 min. The reaction was stopped by the addition of a 100-fold volume of cold methanol. The samples then were applied to a TLC plate and developed and visualized as described above. The band for the product, NBD-diacylglycerol (NBD-DG), on the TLC plate was quantified by densitometry using Image J (NIH). The signal for mutant MCR-1 enzyme was normalized to that for the wild-type MCR-1 enzyme.
