# Supplementary material for: Deep Mutational Scanning Reveals the Active-Site Sequence Requirements for the Colistin Antibiotic Resistance Enzyme MCR-1
Source: mBio. 2021 Nov 16;12(6):e02776-21. doi: 10.1128/mBio.02776-21 (PMC8593676; doi:10.1128/mBio.02776-21)
Supplement: FIG S1 [file mbio.02776-21-sf001.pdf]

	A109		
	**Naïve**	**Colistin**	**PolymyxinB**
**A**	**21476**	**34327**	**57442**
**C**	29575	56831	66789
**D**	17620	5928	11064
**E**	20461	3583	6728
**F**	24617	62403	46849
**G**	36587	70723	66824
**H**	20693	4030	7540
**I**	18805	17895	36892
**K**	27409	4325	21629
**L**	23614	48146	50822
**M**	33202	87116	69294
**N**	31920	11280	18617
**P**	24516	7698	13481
**Q**	33190	7027	23941
**R**	25825	24228	13877
**S**	24788	31434	59379
**T**	31413	45236	60924
**V**	24855	54684	52278
**W**	27226	12167	54615
**Y**	25814	5334	20448
**Total**	520006	594395	594395

A

	T285		
	**Naïve**	**Colistin**	**PolymyxinB**
**A**	29100	3101	5011
**C**	18537	489	966
**D**	32962	990	731
**E**	54778	3451	3492
**F**	29900	1026	1773
**G**	48268	1260	1813
**H**	15473	497	1144
**I**	42519	1074	1724
**K**	42087	11387	12254
**L**	23370	679	928
**M**	33695	1239	3231
**N**	27302	504	1332
**P**	30426	5708	7548
**Q**	30242	1150	2086
**R**	39492	7389	7932
**S**	26007	6128	6586
**T**	**39822**	**526178**	**640251**
**V**	30303	902	1043
**W**	37958	993	1279
**Y**	18709	593	447
**Total**	650950	574738	701571

B

Fig. S1. Representative deep sequencing results of clones from naive and polymyxin-selected MCR-1 libraries. A total of 69 experiments from 23 libraries were sequenced and the results from two libraries (Ala109 and Thr285) are shown in panels A and B. The number of occurrences of the wild-type residue is highlighted in bold for each experiment.
