# Supplementary material for: Deep Mutational Scanning Reveals the Active-Site Sequence Requirements for the Colistin Antibiotic Resistance Enzyme MCR-1
Source: mBio. 2021 Nov 16;12(6):e02776-21. doi: 10.1128/mBio.02776-21 (PMC8593676; doi:10.1128/mBio.02776-21)
Supplement: FIG S2 [file mbio.02776-21-sf002.pdf]

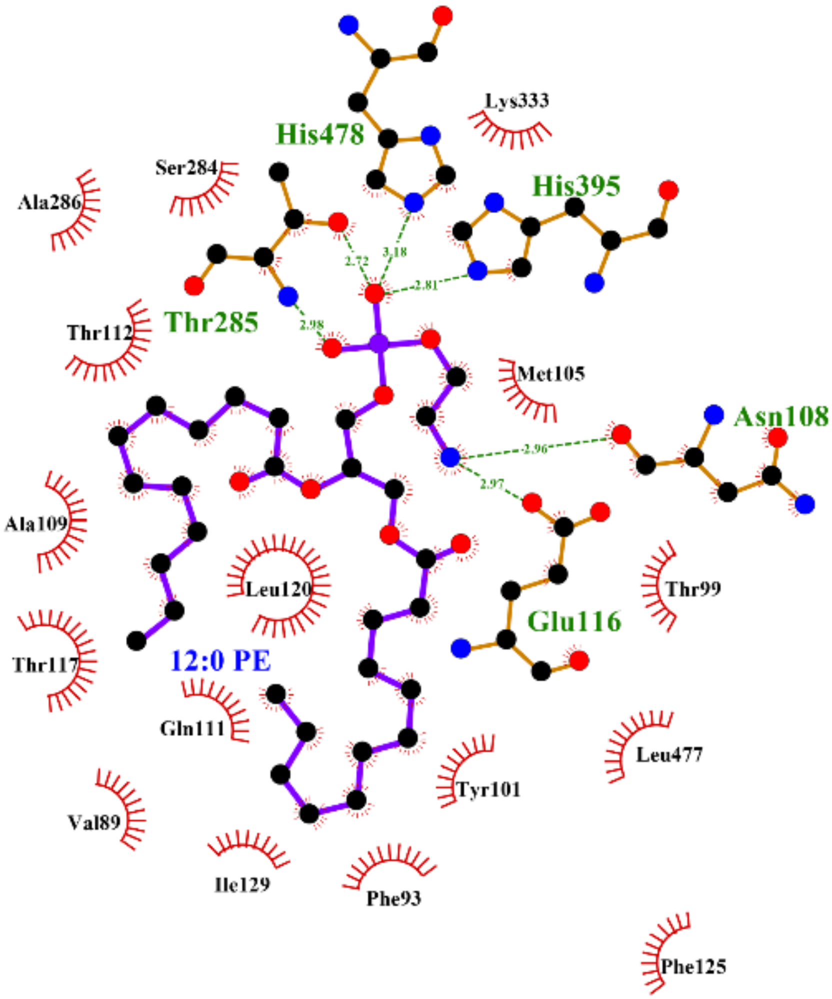

Fig. S2. Ligand-protein interactions in the phosphatidylethanolamine (PE) docking model depicted using LIGPLOT. The 12:0 PE molecule is shown as purple stick and MCR-1 active site residues are shown as orange sticks. Atoms are colored by type (C, black; N, blue; O, red; P, purple). Hydrogen bonding interactions are shown as dashed green lines and the length of hydrogen bonds (Å) is labeled. Ligand-protein hydrophobic contacts are shown as red semicircles.
