# Supplementary material for: Deep Mutational Scanning Reveals the Active-Site Sequence Requirements for the Colistin Antibiotic Resistance Enzyme MCR-1
Source: mBio. 2021 Nov 16;12(6):e02776-21. doi: 10.1128/mBio.02776-21 (PMC8593676; doi:10.1128/mBio.02776-21)
Supplement: FIG S3 [file mbio.02776-21-sf003.pdf]

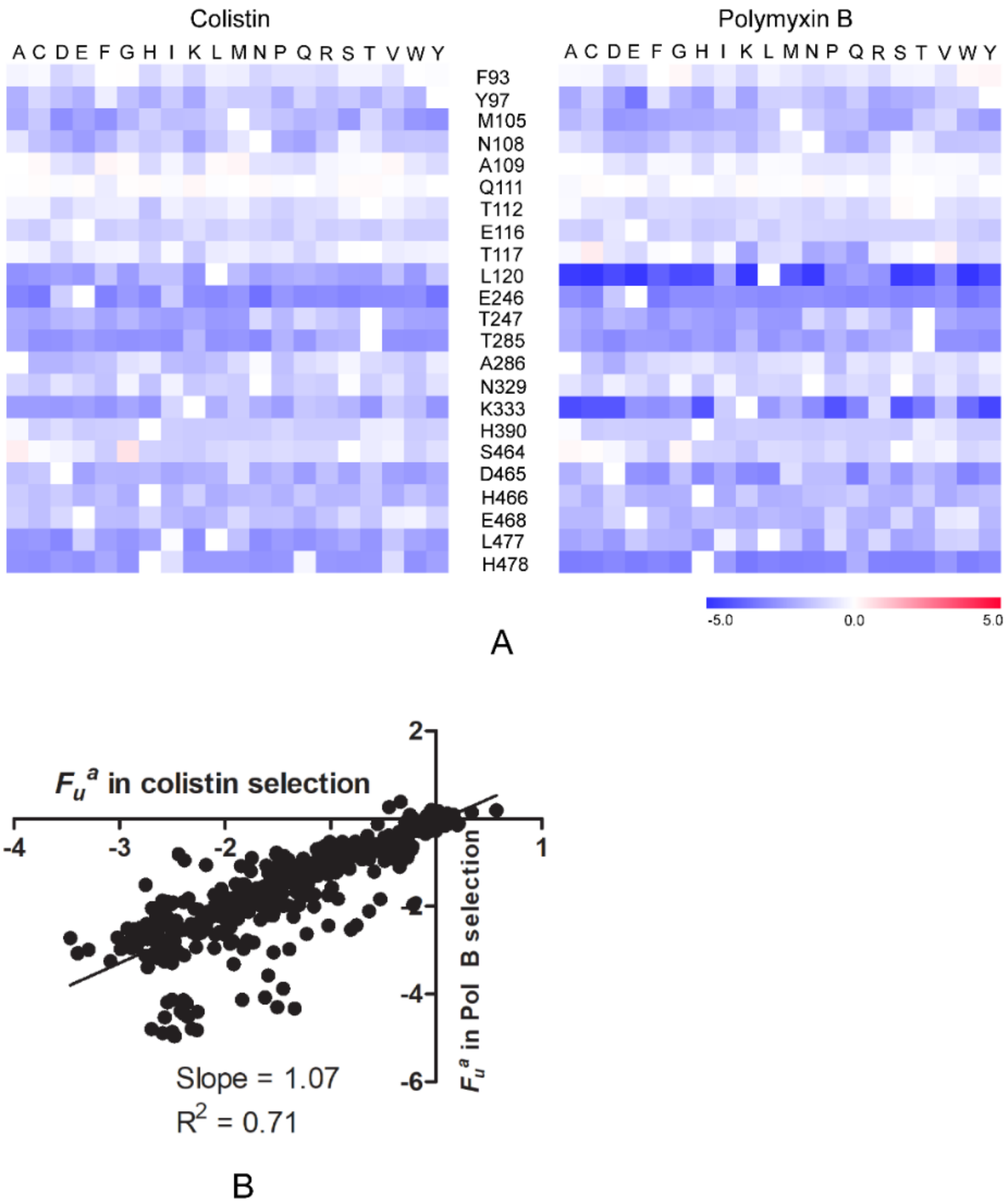

Fig. S3. Relative fitness of MCR-1 mutants under selection with colistin or polymyxin B. **A**. Heat-map representation of fitness effect of amino acid substitutions at each position under selection with colistin or polymyxin B. The relative fitness (F_u^a) was calculated based on the frequency of occurrence of the mutant and wild-type allele from the deep sequencing data for the naïve libraries and those selected by colistin or polymyxin B as described in Materials and Methods. The correspondence between heat map colors and F_u^a values is shown below the map. On the map, blue color represents a negative fitness effect, red color represents a positive fitness effect, and white represents neutral fitness effect. **B**. Relationship between the relative fitness of MCR-1 mutants in colistin selected libraries versus polymyxin B selected libraries. Linear regression analysis was performed for the F_u^a of each mutant in the colistin selected libraries plotted versus the F_u^a of the corresponding mutant in the polymyxin B selected libraries.
