# Supplementary material for: Deep Mutational Scanning Reveals the Active-Site Sequence Requirements for the Colistin Antibiotic Resistance Enzyme MCR-1
Source: mBio. 2021 Nov 16;12(6):e02776-21. doi: 10.1128/mBio.02776-21 (PMC8593676; doi:10.1128/mBio.02776-21)
Supplement: FIG S4 [file mbio.02776-21-sf004.pdf]

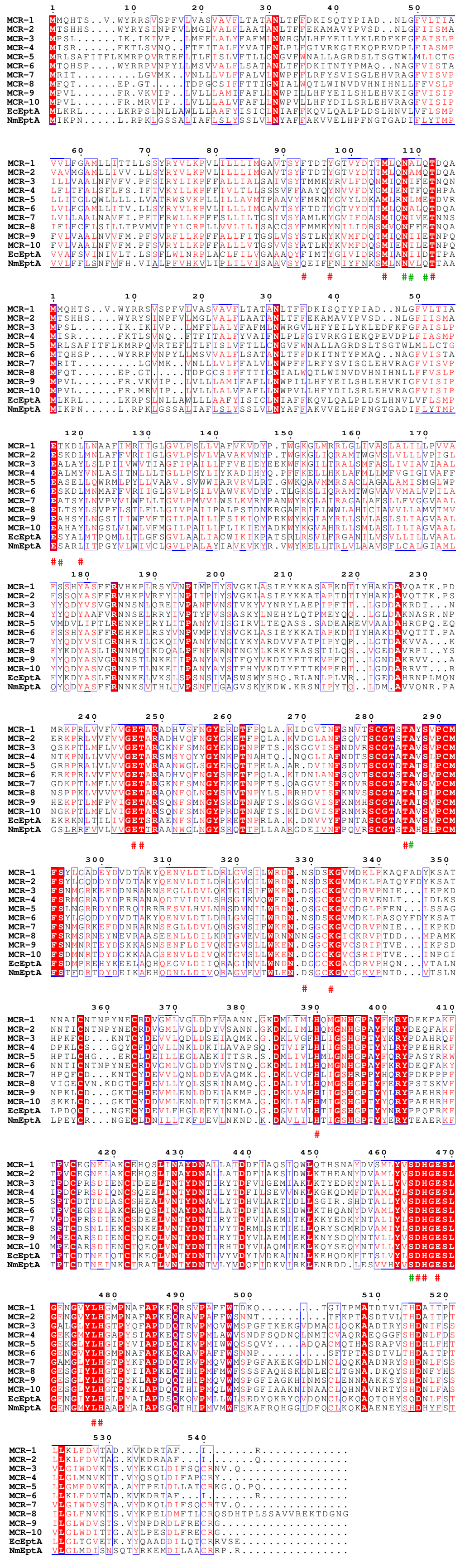

Fig. S4. Multiple sequence alignment among MCR homologues and chromosomal phosphoethanolamine transferases from *E. coli* (EcEptA) and *N. meningitidis* (NmEptA) using T-Coffee and ESPript 3. The 23 residue positions of MCR-1 for which randomization libraries were constructed are marked by # underneath the residues with red and green colors for essential and non-essential residues, respectively.
